# Identification of pyroptosis‐related gene prognostic signature in head and neck squamous cell carcinoma

**DOI:** 10.1002/cam4.4825

**Published:** 2022-05-16

**Authors:** Zhanzhan Li, Lin Shen, Yanyan Li, Liangfang Shen, Na Li

**Affiliations:** ^1^ Department of Oncology Xiangya Hospital, Central South University China; ^2^ Department of Nursing Xiangya Hospital, Central South University China; ^3^ National Clinical Research Center for Geriatric Disorders Xiangya Hospital, Central South University China

**Keywords:** GEO, head and neck squamous cell carcinoma, prognosis, Pyroptosis, TCGA

## Abstract

**Background:**

Head and neck squamous cell carcinoma (HNSCC) is a life‐threatening disease with poor prognosis. Pyroptosis has been recently disclosed as a programmed cell death triggered by invasive infection, involved in cancer development. However, the prognosis role of pyroptosis‐related genes in HNSCC has not been discussed.

**Methods:**

The RNA sequence data of pyroptosis‐related genes were obtained from The Cancer Genome Atlas (TCGA) database. Cox regression and the least absolute shrinkage and selection operator (LASSO) analysis were performed to screen the HNSCC survival‐related signature genes. We established a HNSCC risk model with the identified prognostic genes, then divided the HNSCC patients into low‐ and high‐risk subgroups according to median risk score. Moreover, we utilized Gene Expression Omnibus (GEO) dataset to validate the risk model. Go and KEGG analyses were conducted to reveal the potential function of differential expression of genes that identified between low‐ and high‐risk subgroups. ESTIMATE algorithm was performed to investigate the immune infiltration of tumors. Correlation between signature gene expression and drug‐sensitivity was disclosed by Spearman's analysis.

**Results:**

We constructed a HNSCC risk model with identified seven pyroptosis‐related genes (*CASP1, GSDME, IL6, NLRP1, NLRP2, NLRP6*, and *NOD2*) as prognostic signature genes. High‐risk subgroup of HNSCC patients in TCGA cohort correlated with lower survival probability than patients from low‐risk subgroup (*p* < .001), and the result is verified with GEO dataset. In addition, 161 genes were identified differentially expressed between the low‐ and high‐risk subgroups in the TCGA cohort, mainly related to immune response. Higher PD‐L1 expression level was found in the high‐risk subgroup that indicated the possible employment of immune checkpoint inhibitors. *IL6* was positively correlated with WZ3105 and MPS‐1‐IN‐1 in the cancer therapeutics response portal database.

**Conclusion:**

We built and verified a risk model for HNSCC prognosis using seven pyroptosis‐related signature genes, which could predict the overall survival of HNSCC patients and facilitate treatment.

## Graphical abstract

We built and verified a risk model for HNSCC prognosis using 7 pyroptosis‐related signature genes, which could facilitate the implementation of the HNSCC treatment.

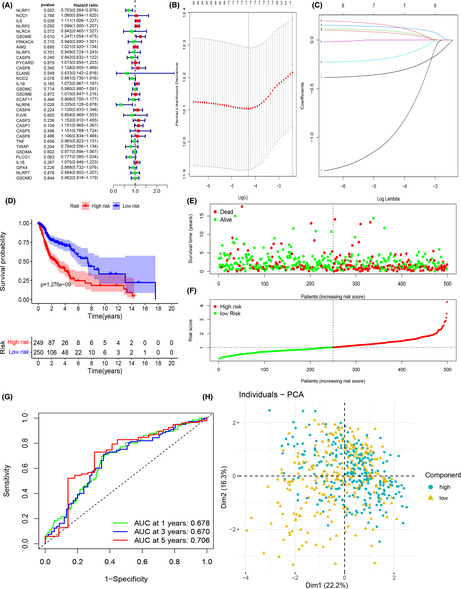

## 1 INTRODUCTION

HNSCC comprises around 90% head and neck cancer, is an aggressive and common disease with an estimated 890,000 new cases and 450,000 deaths in 2018. The incidence rate of HNSCC continues to climb annually and would increase by 30% by 2030.^1^, ^2^ The main treatment options for HNSCC are surgery and radiation with or without chemotherapy, while managing recurrent tumors require multimodality treatment including application of immune inhibitors.^3^ In spite of advances in therapies, the 5‐year survival rate of HNSCC patients remains 40–50%,^1^, ^4^ the prognosis is frustrating. Thus, establishing a reliable prognostic model may assist to guiding clinical treatments for HNSCC patients.

Pyroptosis is a newly discovered programmed cell death triggered by invasive infection, and it plays an important role in pathogen clearance. Morphologically, pyroptosis results in cell swelling, plasma membrane rupture, chromatin fragmentation, leading to intracellular release of pro‐inflammatory factors including IL1b, IL18, and other cellular contents.^5^, ^6^ Pyroptosis can be induced via a canonical pathway with the activation of CASP1 and a noncanonical pathway with the activation of CASP4/5/11, and then a key protein gasdermin D (GSDMD) is cleaved, oligomerized and transported to membranes. Eventually, there are pores start to form in the cell membrane, leading to the secretion of cytokines and cell rupture.^7^, ^8^ Accumulating evidences elaborate the close relationship of pyroptosis and cancers. Pyroptotic tissues may release the inflammatory mediators and cause the chronic inflammation which increases the risk of cancer.^9^, ^10^ For example, secreted high‐mobility group box protein 1 (HMGB1) induces colitis‐associated colorectal cancer (CAC) proliferation through ERK1/2 pathway.^11^ On the contrary, pyroptosis may also inhibit the tumor development. Higher expression of NAcht leucine‐rich repeat protein 1 (NALP1) which mediates inflammasome activation, correlates with lower risk of metastasis, and longer survival of colon cancer patients.^12^ Downregulation of GSDMD slows down tumor proliferation and predicts a good prognosis in non‐small cell lung cancer (NSCLC).^13^ Therefore, a better understanding of the molecular profiling and mechanism in different cancers would provide more information on clinical prognosis and treatments.

Considering the important role of pyroptosis in tumor development, recent researches identify the novel pyroptosis‐related gene signatures for diagnose or prognosis of lung adenocarcinoma,^14^ ovarian cancer,^15^ gastric cancer,^16^ and skin cutaneous melanoma.^17^ However, less studies are found in HNSCC. Hence, we applied the bioinformatic analysis to disclose the expression levels of pyroptosis‐related gene in HNSCC and adjacent normal tissues, and evaluate the prognostic values of these genes, and investigate the relationships between pyroptosis and immune response in tumor microenvironment (TME).

## 2 MATERIALS AND METHODS

### 2.1 Data sources

The RNA sequence (RNA‐seq) data of 502 HNSCC patients and 44 adjacent normal tissues, and corresponding clinical parameters were obtained from TCGA database on July 23, 2021. The RNA‐seq data of 97 HNSCC patients and clinical parameters were obtained from GEO database (ID: GSE41613).

### 2.2 Differentially expressed profile of pyroptosis‐related genes

In total 33 pyroptosis‐related genes were extracted from prior studies (Table S1).^15^ We distinguished the differentially expressed pyroptosis‐related genes between HNSCC and adjacent normal tissues via “limma” and “reshape2” packages. We uploaded the identified protein names to functional protein association networks STRING (https://cn.string‐db.org/), and retrieved the protein–protein interaction (PPI) profile.

### 2.3 Establishment and validation of prognostic signature

To develop the prognostic model, we first conducted univariate Cox regression and LASSO analysis^18^ using the R package “glmnet” to filter the candidate genes, and we used the minimum parameters to confirm the penalty factor *λ*. Next, we adopted the following equation to calculate the risk score: Risk Score = Gene1_*CoefixExpi*_ + Gene2 _*CoefixExpi*_ + …GeneN _*CoefixExpi*_ (*Coef*: coefficients, *Exp*: gene expression level). Five hundred and two HNSCC patients from TCGA dataset were divided into low‐ and high‐risk subgroups based on the calculated median risk score. Subsequently, we compared the overall survival curves between the subgroups via Kaplan–Meier analysis, and we also described 1‐, 3‐, and 5‐year overall survival by means of time‐dependent receiver operating characteristic (ROC) analysis. Area under the ROC curve (AUC) curve is applied to evaluate the predictive power of the model. We prepared the principal component analysis (PCA) plot using the “prcomp” function in the “stats” R package. The nomogram model was constructed on the basis of risk score and clinical information (age, gender, grade, and stage) to predict the overall survival of HNSCC patients.

### 2.4 Independent prognostic analysis of risk score

We downloaded the clinical information (age, gender, grade, and stage) of patients separately from the TCGA and GEO cohort, and conducted univariate and multivariable Cox regression to analyze the independent prognostic characteristics of these variables and risk score in the model.

### 2.5 Functional enrichment analysis

We first screened 161 differentially expressed genes between low‐ and high‐risk HNSCC subgroups according to the criteria (|log_2_FC| > 1.5 and FDR < 0.001). Next, we performed Geno ontology (Go) and Kyoto Encyclopedia of Genes and Genomes (KEGG) analyses.

### 2.6 Immune infiltration, gene variation, and drug‐sensitivity analysis

To investigate the immune infiltration status of the tumors, we used the Estimation of STromal and Immune cells in MAlignant Tumor tissues using Expression data (ESTIMATE) algorithm^19^ to analyze immune components and overall stroma in low‐ and high‐risk subgroups in the TCGA cohort. Gene variations were compared between subgroups using maftools. Correlation between signature gene expression and drug‐sensitivity was disclosed by Spearman's analysis in the cancer therapeutics response portal database.

### 2.7 Statistical analysis

We applied Student *t*‐test and one‐way ANOVA to compare the differences of continuous variables between the HNSCC and normal tissues. Chi‐squared test was applied to compare the categorical variables. The survival curves of Kaplan–Meier analysis were compared by the log‐rank test. The hazard ratio (HR) and 95% confidence interval (CI) of pyroptosis‐related genes and clinical parameters were calculated along with the application of univariate and multivariate Cox regression. R software (v4.0.2) was used to perform the statistical analysis. *p* < 0.05 was considered significant level.

## 3 RESULTS

### 3.1 Identification of differential pyroptosis‐related genes in HNSCC

**FIGURE 1:**
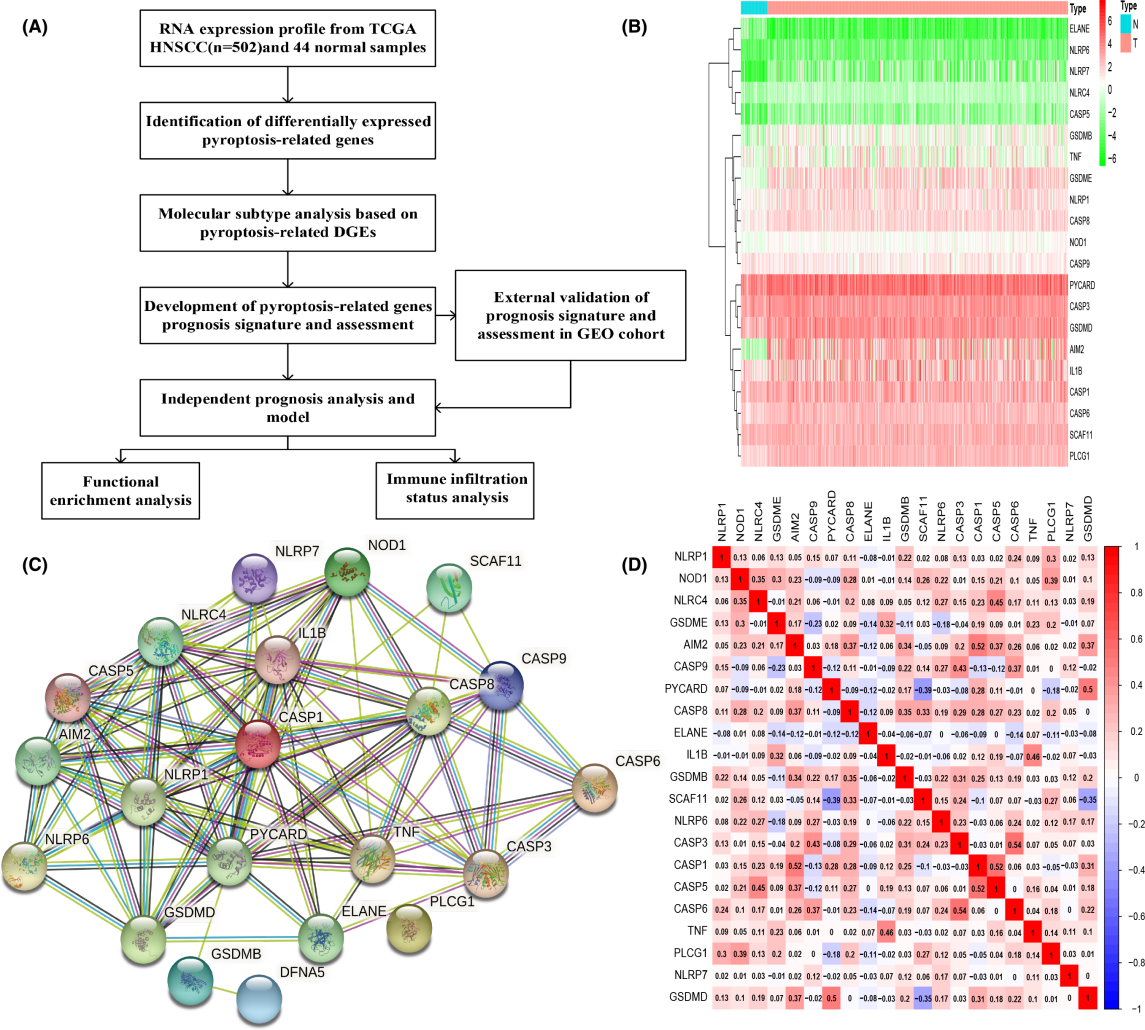
Identification of differentially expressed pyroptosis‐related genes. (A) The flow chart of data analysis. (B) Heatmap revealed that 21 pyroptosis‐related genes expressed differently between HNSCC and normal samples. (C) PPI network of 21 differentially expressed genes. (D) Correlation plot of 21 differentially expressed genes

We first compared 33 pyroptosis‐related gene expressions in TCGA HNSCC dataset (*n* = 502) and normal samples (*n* = 44), followed by identification and analysis. The workflow was shown in Figure 1A. We had identified 21 genes that displayed different expression levels in the tumor group versus normal group, and the RNA levels of these genes were presented as heatmap (adjusted *p* < 0.05, Figure 1B). Next, we performed PPI analysis in order to reveal the potential interaction network of these 21 pyroptosis‐related genes (Figure 1C), and we found *AIM2, CASP1, CASP5, CASP8, GSDMD, IL1B, PYCARD, NLRC4,* and *NLRP1,* were hub genes. The correlation analysis of these 21 pyroptosis‐related genes was shown in Figure 1D.

### 3.2 Cluster analysis based on pyroptosis‐related genes

**FIGURE 2:**
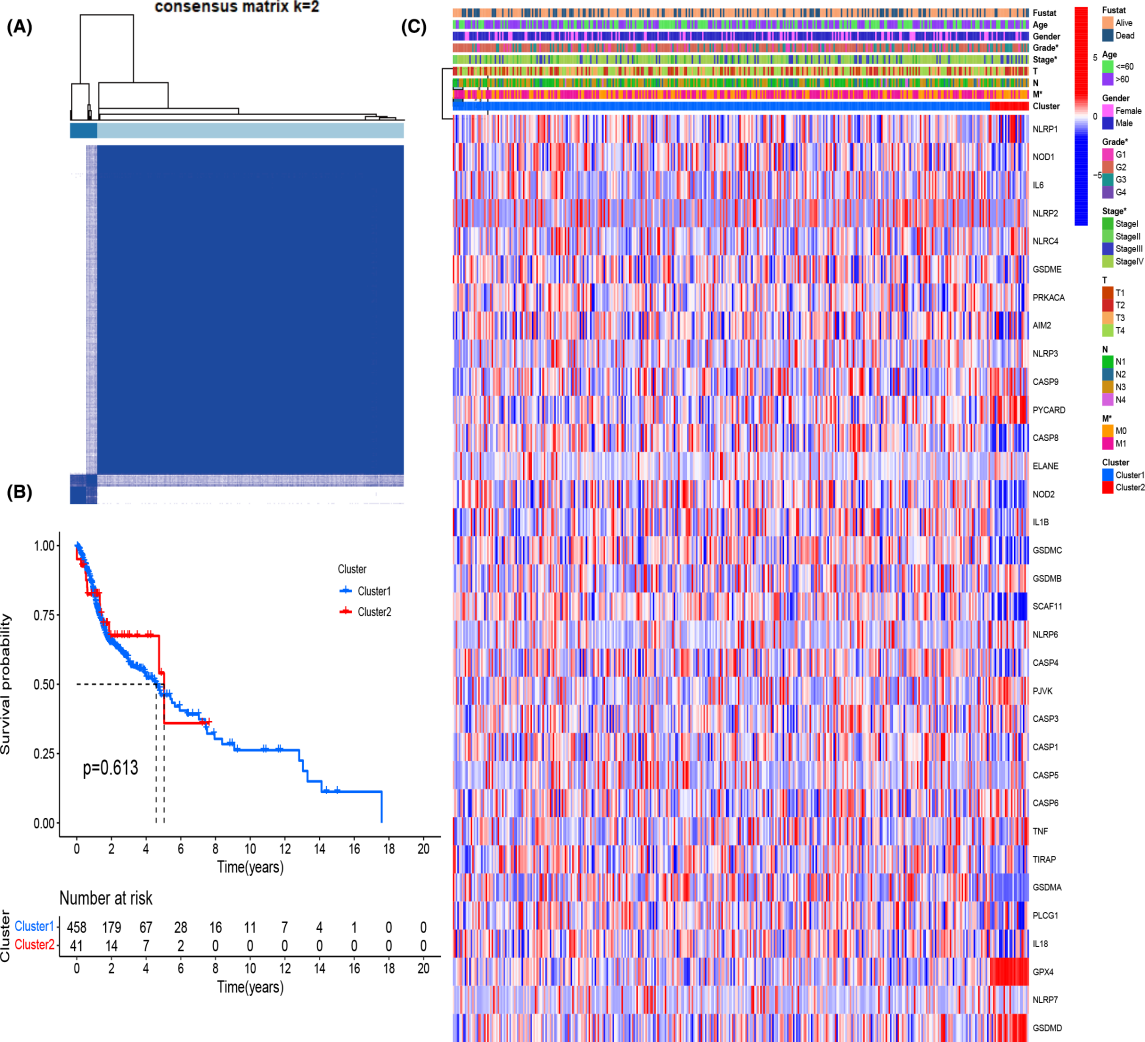
Molecular subtype based on differentially expressed pyroptosis‐related genes. (A) Cluster analysis (*k* = 2). (B) Kaplan–Meier curves of two clusters. (C) Heatmap displayed the expression levels of 33 pyroptosis‐related genes and the clinical characteristics of two clusters

To reveal the potential connections of 21 differentially expressed pyroptosis‐related genes to HNSCC subtypes, we applied k‐means consensus clustering method to analyze 502 HNSCC patients in TCGA dataset. We found that when the clustering variable *k* = 2, the points in the same group are similar, and dissimilar in different groups, which mean the 502 HNSCC patients could be separated into two clusters on the basis of these 21 differentially expressed genes (Figure 2A). Then, the probability of survival in given length of time was compared between these two clusters using Kaplan–Meier survival curve (Figure 2B), but no differences were observed (*p* = .613). A total of 33 pyroptosis‐related gene expressions and the clinical characteristics including age (≤60 or >60), gender (female or male), tumor differentiation (G1‐G3), tumor node metastasis classification (TNM), and survival status (alive or dead) were displayed in the heatmap (Figure 2C), but no obvious differences were seen.

### 3.3 Development of prognostic biomarkers of pyroptosis‐related genes

**FIGURE 3:**
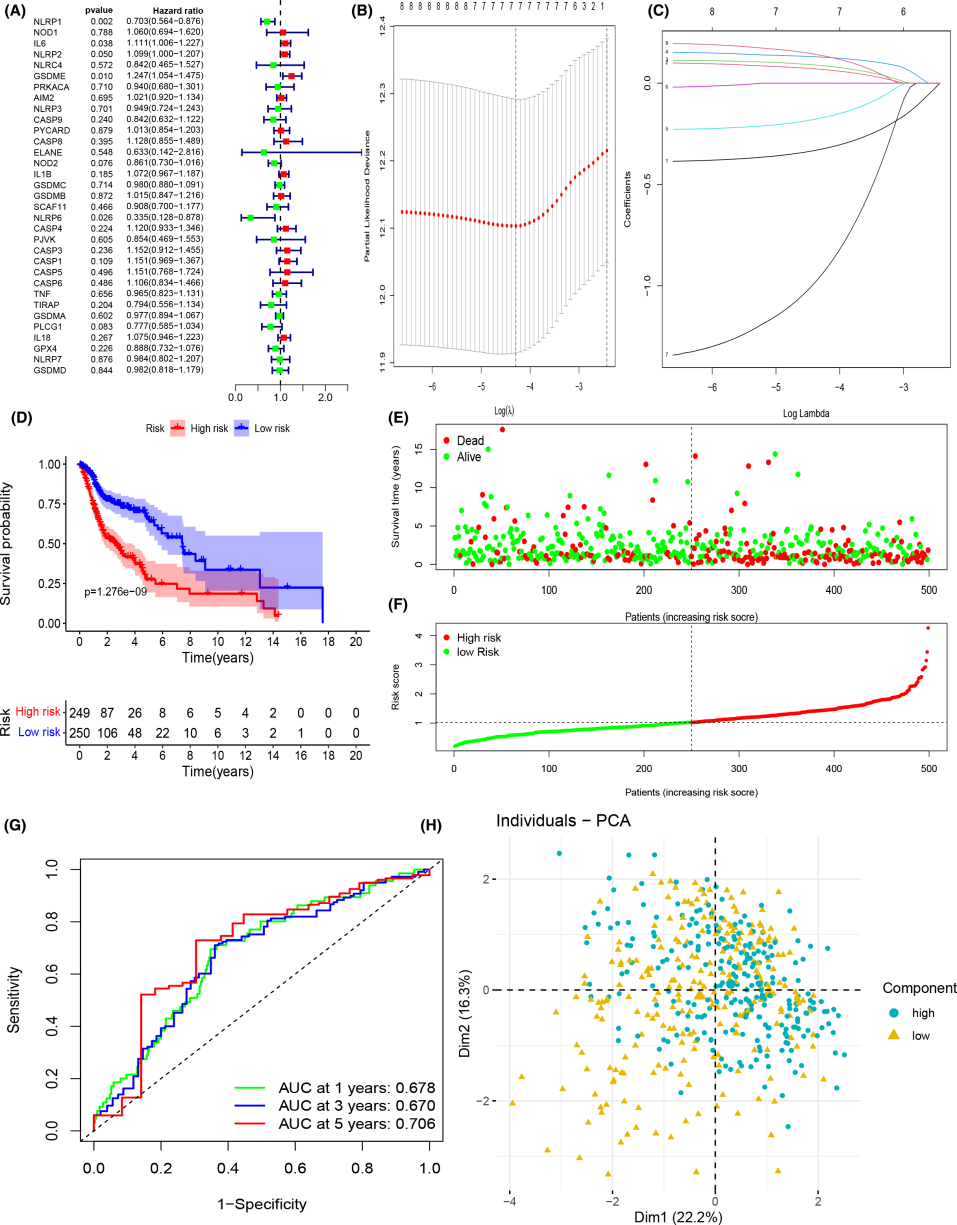
Development and assessment of prognostic signature based on pyroptosis‐related genes in the TCGA cohort. (A) Forest of univariate Cox regression‐related overall survival prognosis. (B) Cross‐validation for identifying parameters in the LASSO. (C) LASSO regression identified seven prognosis‐related genes. (D) Kaplan–Meier curves of low‐ and high‐risk subgroups based on risk score. (E) Scatterplot of relationship between risk scores and survival time/survival outcomes. (F) Risk score of HNSCC patients were presented based on the low‐ and high‐risk subgroups. (G) ROC curves of prognostic signature in HNSCC patients. H PCA analysis of patient distributions from low‐ and high‐risk group

To evaluate the effect of 33 pyroptosis‐related genes on survival of HNSCC patients, univariate Cox regression and the LASSO analysis were applied (Figure 3A‐C). Up to seven genes (*CASP1, GSDME, IL6, NLRP1, NLRP2, NLRP6*, and *NOD2*) were selected as survival‐related signature genes according to the optimum penalty factor *λ* value. Among them, *NLRP1, NLRP6,* and *NOD2* were regarded as good prognostic biomarkers (HR < 1), while *IL6, NLRP2, GSDME,* and *CASP1* were bad prognostic biomarkers (HR > 1). A HNSCC risk model consisting of these seven prognosis‐related genes was generated (risk score = (0.197 × *CASP1* exp.) + (0.145 × *GSDME* exp.) + (0.096 × *IL6* exp.) + (−0.392 × *NLRP1* exp.) + (0.116 × *NLRP2* exp.) + (−1.359 × *NLRP6* exp.) + (−0.237 × *NOD2* exp.)). We further investigated the survival status of the HNSCC patients by using Kaplan–Meier method on this risk model. In total 502 HNSCC patients were equally divided into two subgroups according to the median risk score, and it was observed that patients in high‐risk subgroup apparently correlated to shorter survival time (*p* < .001, Figure 3D–F). Then, we measured the ability of our constructed model by means of time‐dependent ROC method to predict future risk. As shown in Figure 3G, AUC for 1‐, 3‐, and 5‐year survival were 0.678, 0.670, and 0.706 separately. The distribution of patients in two subgroups was shown in the PCA plot (Figure 3H).

**FIGURE 4:**
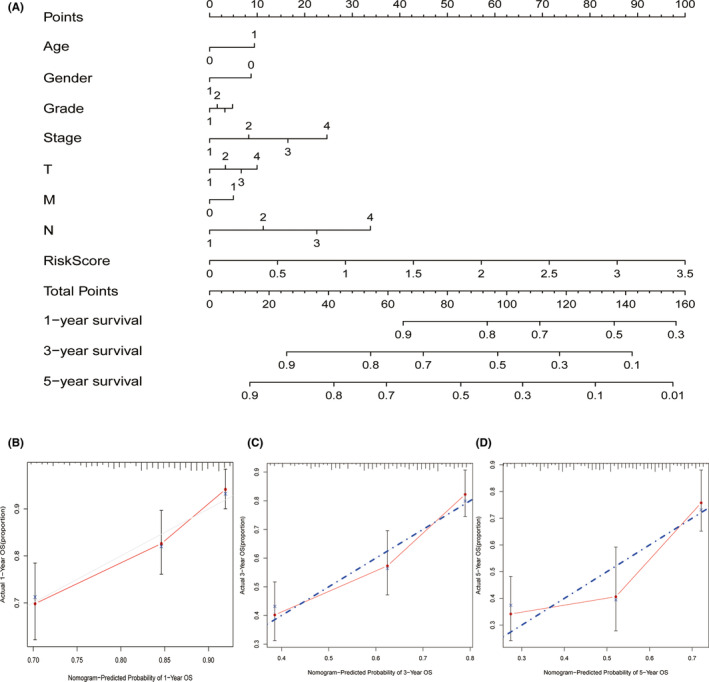
Establishment of a predictive nomogram. (A) Nomogram to predict 1‐, 3‐, and 5‐year survival rate of HNSCC patients. (B) Calibration curves of the nomogram predicting survival rate of HNSCC patients in the validation cohort (x‐axis: predicted survival probabilities; y‐axis: actual observed survival probabilities)

Furthermore, we built a predictive nomogram consisting of clinical parameters and risk score to predict the 1‐, 3‐, and 5‐year survival rates of HNSCC patients (Figure 4A). The calibration curves of the nomogram indicated the relatively good prediction of overall survival rates (Figure 4B–D). In addition, the predictive ability of model and the clinical characteristics of patients were shown in Figure S1.

### 3.4 Validation and clinical value of prognostic signature

**FIGURE 5:**
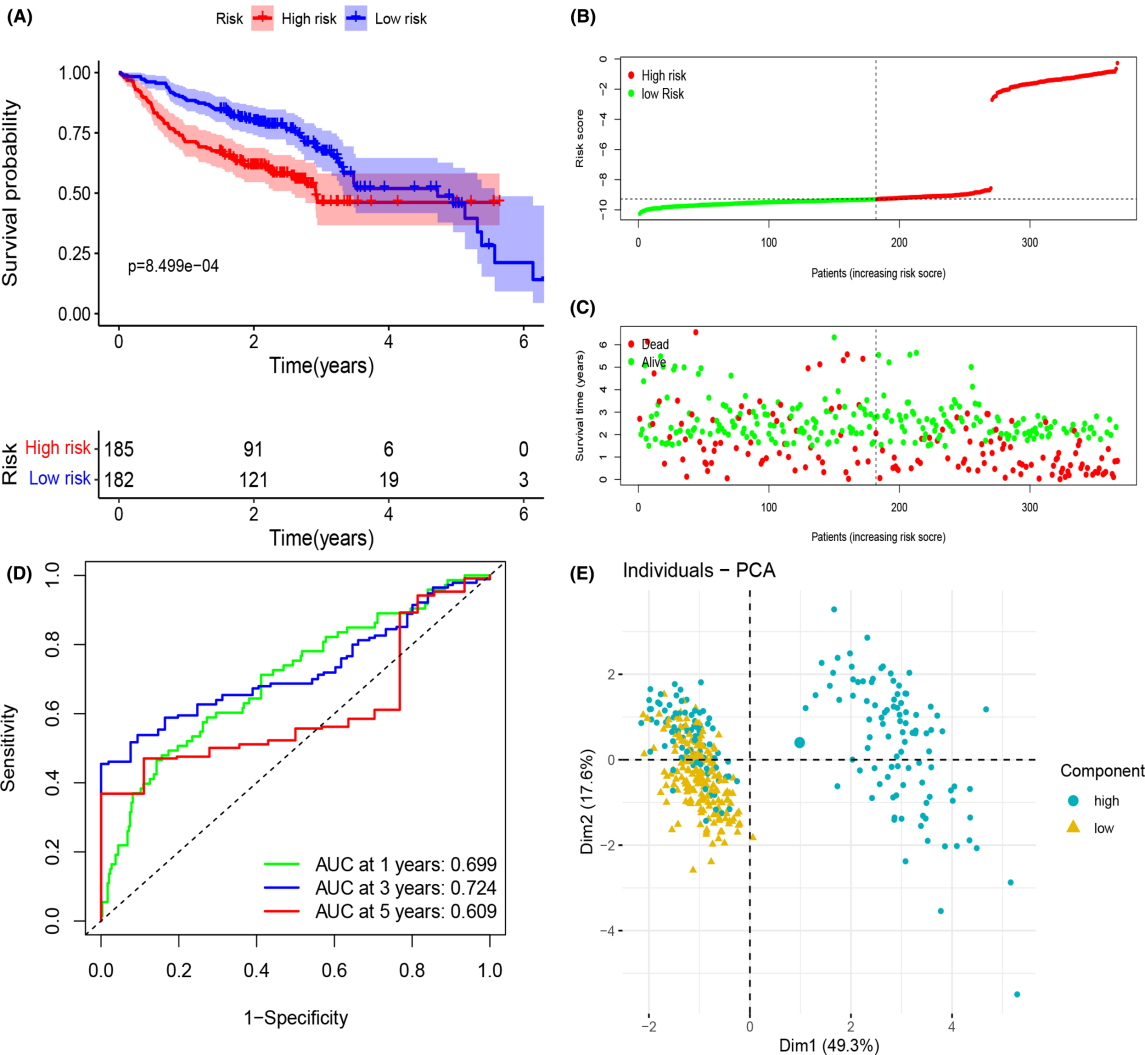
External validation of prognostic signature genes in the GEO cohort. (A) Kaplan–Meier curves of low‐ and high‐risk subgroups based on risk score. (B) Risk score of HNSCC patients was presented. (C) Scatterplot of relationship between risk scores and survival time/survival outcomes. (D) ROC curves of prognostic signature in HNSCC patients. (E) PCA analysis of patient distributions from low‐ and high‐risk subgroup

GEO dataset was utilized to validate our established model. Based on the calculated median risk score, the 97 HNSCC patients from the dataset (GSE41613) were divided into two subgroups, and it was obviously seen that patients in the high‐risk subgroup had lower probability of survival and shorter survival time when compared to the patients in the low‐risk subgroup (*p* < .001, Figure 5A‐C). Moreover, time‐dependent ROC curve disclosed that AUC for 1‐, 3‐, and 5‐year survival were, 0.699, 0.724, and 0.609 separately (Figure 5D), which was consistent to our previous analysis with TCAG dataset, exhibiting a good prediction using our model. The PCA plot of two subgroups was shown in Figure 5E. Considering limited data of GEO were available to verify our model, we had conducted internal validation by dividing patients from TCGA into train set and validate set to measure the accuracy of our model, and the results were good and shown in Figure S2.

### 3.5 Independent prognostic analysis for risk score

**FIGURE 6:**
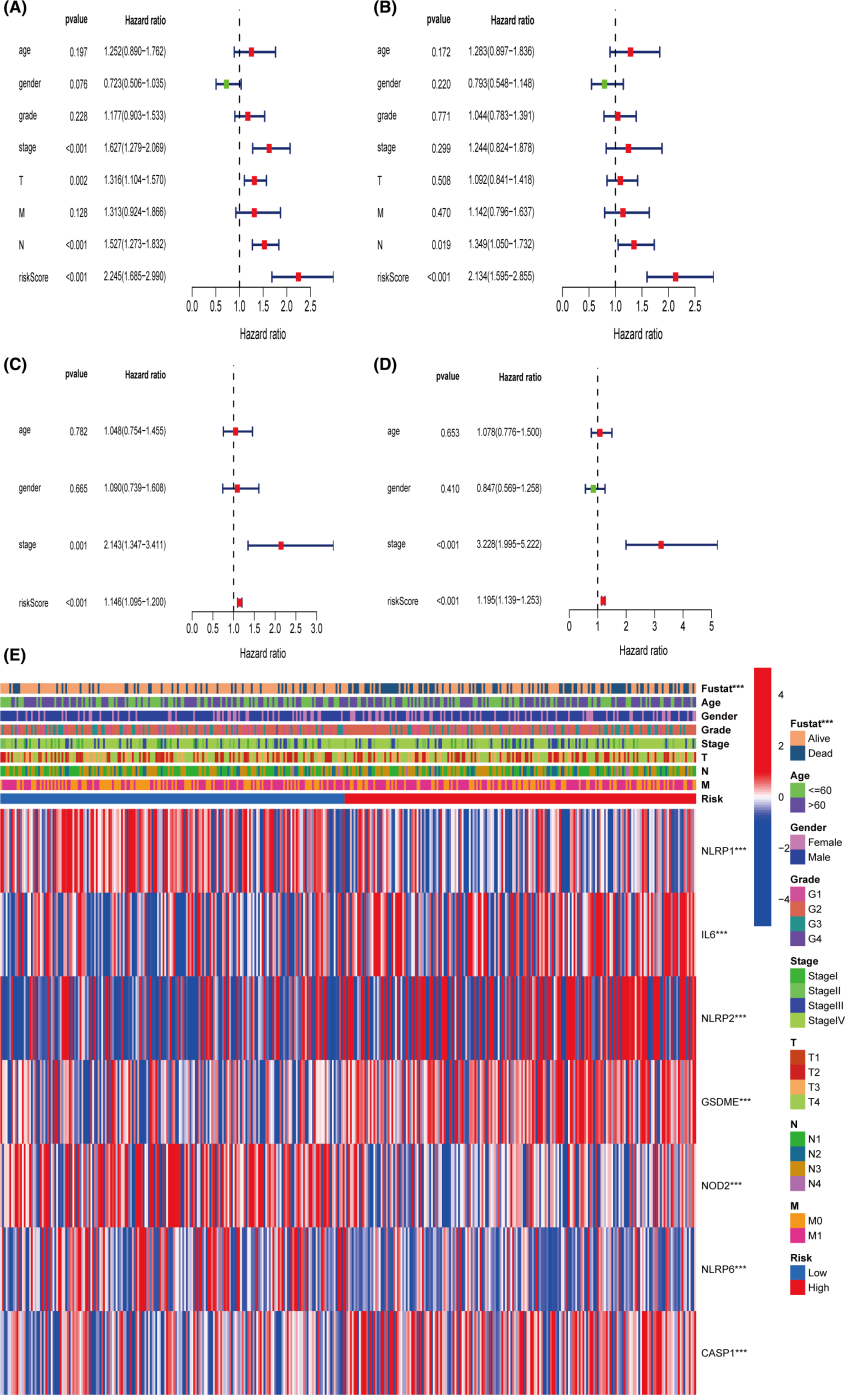
Independent prognostic analysis for risk score. (A) Univariate Cox regression analysis of risk score in the TCGA cohort. (B) Multivariate Cox regression analysis of risk score in the TCGA cohort. (C) Univariate Cox regression analysis of risk score in the GEO cohort. (D) Multivariate Cox regression analysis of risk score in the GEO cohort. (E) Heatmap for association between clinical parameters and identified prognostic signature genes

To confirm the risk score determined by the seven‐gene signature model in prognosis could act as an independent prognostic factor, we employed univariate and multivariate Cox regression analysis. In TCGA cohorts, both analyses verified that the risk score was independent for prediction of poor survival in HNSCC patients (univariate analysis, HR = 2.245 95% CI: 1.685–2.990 and multivariate analysis, HR = 2.134 95% CI: 1.595–2.855, Figure 6A, B), and in GEO cohort, the same conclusion was drawn (univariate analysis, HR = 1.146 95% CI: 1.095–1.200 and multivariate analysis, HR = 1.195 95% CI: 1.139–1.253, Figure 6C, D). A heatmap of clinical characteristics and signature genes for the TCGA cohort was generated in Figure 6E, and we discovered that expressions of all seven signature genes as well as the survival status (alive or dead) had statistical differences between the two subgroups.

### 3.6 Functional enrichment analysis indicates the different pathways in low‐ and high‐risk subgroups

**FIGURE 7:**
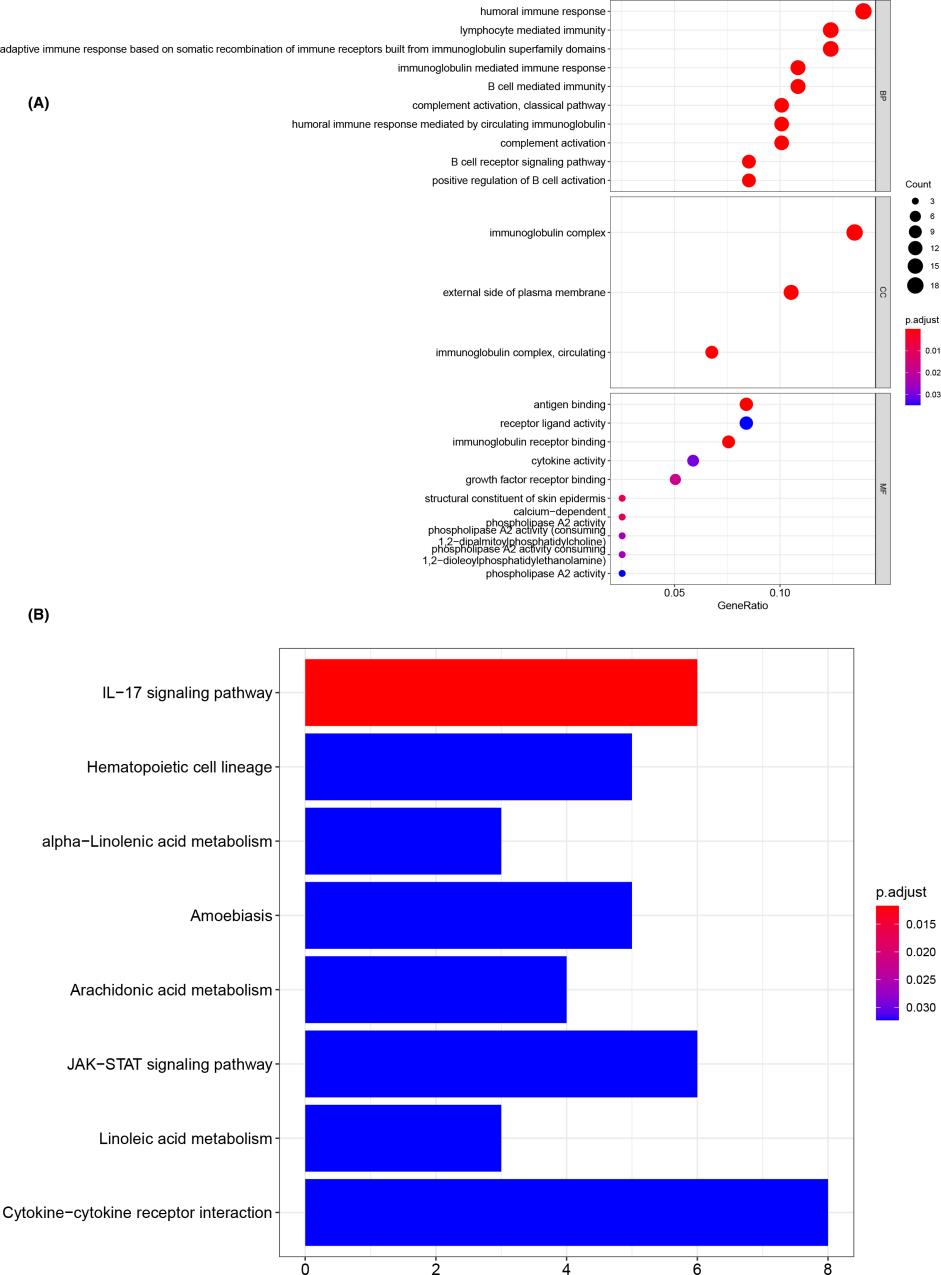
Functional enrichment analysis of identified differential expressed genes between low‐ and high‐risk subgroups. (A) Bubble plot for GO analysis. B Barplot of KEGG pathway analysis

To further investigate the different mechanisms that might affect the survival status of patients in two subgroups, we first identified 161 genes that expressed differently between two subgroups in the TCGA cohort, and then performed functional enrichment analysis (Go and KEGG). We found that the low‐ and high‐risk subgroups had diverse relationship with immunity and immune response (Figure 7A, B). Go analysis revealed that these genes enriched in regulation of humoral immune response, lymphocyte‐mediated immunity, and adaptive immune response, etc. While KEGG result identified an important pathway, IL‐17 signaling pathway that has been related to cancer progression.^20^

### 3.7 Immune filtration analysis

**FIGURE 8:**
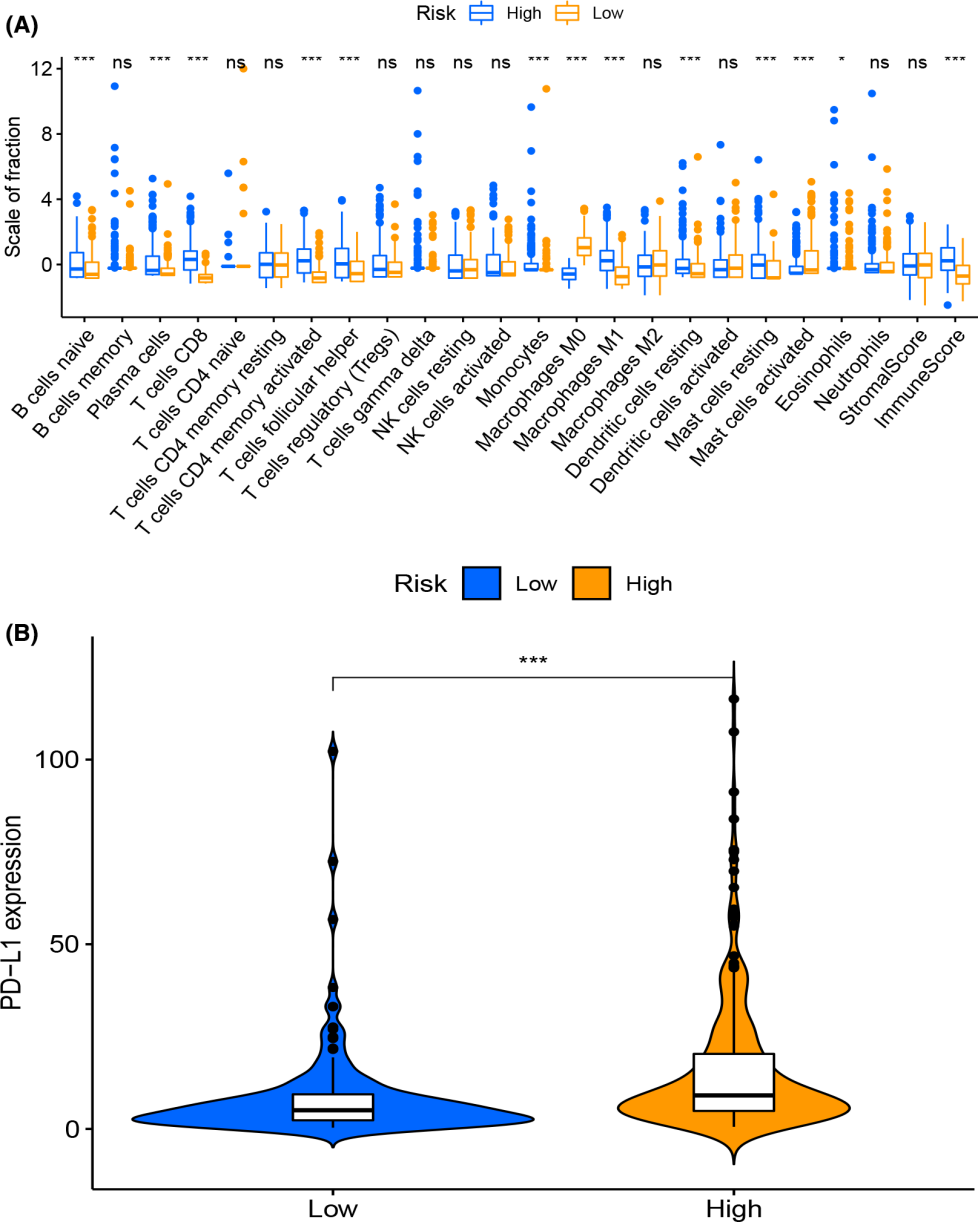
Analysis of immune cell infiltration in low‐ and high‐risk groups. (A) Boxplot for immune cells comparisons between low‐ and high‐risk subgroups. (B) Violin plot for PD‐L1 expression between low‐ and high‐risk subgroups

Next, we used ESTIMATE algorithm to calculate the immune and stromal scores in low‐ and high‐risk subgroups in the TCGA cohort. High‐risk subgroup consisted of especially higher level of naïve B cells, plasma cells, CD8+ T cells, activated memory CD4+ T cells, T follicular helper cells, monocytes, M1 macrophages, resting dendritic cells, and resting mast cells, but lower level of M0 macrophages and activated mast cells. It was shown that high‐risk subgroup had significant higher immune score than that of low‐risk subgroup (Figure 8A), which suggested that in TME, high‐risk subgroup might have more immune cell infiltration. In addition, we also analyzed the expression level of PD‐L1 in two subgroups, and found higher level of PD‐L1 in patients of high‐risk subgroup (Figure 8B), indicating the potential clinical benefit of medication of immune checkpoint inhibitors.

### 3.8 Gene variation and drug‐sensitivity analysis

**FIGURE 9:**
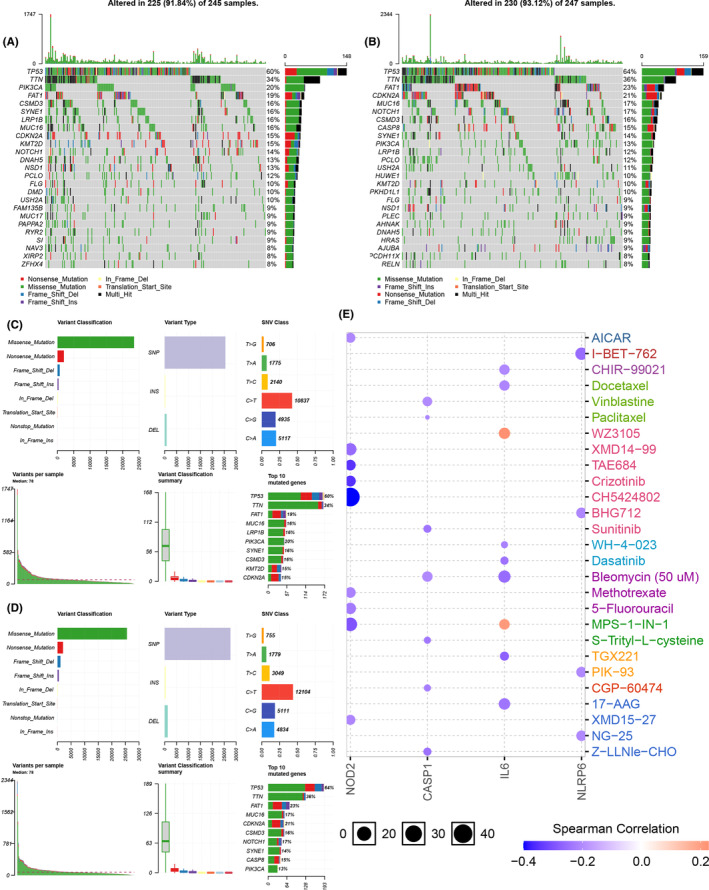
Gene variation and drug‐sensitivity analysis. (A, C) Gene variation analysis in low‐risk subgroup. (B, D) Gene variation analysis in high‐risk subgroup. (E) The correlation between four prognostic pyroptosis‐related genes and drug‐sensitivity

Cancer cells always harbor numerous genome mutations, including gene sequence (single nucleotide polymorphism, nucleotide insertion, nucleotide deletion, and sequence substitution) and structural (copy number variation, sequence inversion, and translocation) variants. A picture of genomic variation profile would provide us with more information on drug screening.^21^ We first analyzed the gene mutations in low‐risk subgroup (Figure 9A, C) and high‐risk subgroup (Figure 9B, D) separately, and not much differences between two subgroups were seen concerning to variant classification and types. However, the mutations frequencies of *CDKN2A*, *NOTCH1*, and *CASP8* were significantly higher in high‐risk subgroup than in low‐risk subgroup. We further investigated if our seven pyroptosis‐related signature genes could serve as biomarkers for drug screening. The result revealed that the expressions of *NOD2, CASP1, IL6,* and *NLRP6* were negatively correlated with some or most drugs, while *IL6* was also positively correlated with WZ3105 and MPS‐1‐IN‐1 in the cancer therapeutics response portal database (Figure 9E).

## 4 DISCUSSION

The abilities to escape from deaths is one of the most essential characters of cancer cells that would probably cause unsatisfactory therapeutic outcomes.^6^ As an inflammatory cell death program, pyroptosis is triggered by various pathological stimuli, and its inflammatory features distinguish it from apoptosis and necroptosis.^22^ Numerous studies have well described the mechanisms of apoptosis and necroptosis, but the role of pyroptosis is little elucidated in HNSCC.

In this study, we first evaluated the RNA expression level of 33 pyroptosis‐related genes in HNSCC and adjacent normal tissues in TCGA cohort, we distinguished 21 genes expressing differently between HNSCC and normal tissues. Using k‐means consensus clustering method, we divided 502 HNSCC patients equally into two subgroups, but no survival differences for patients from two subgroups were observed. To further evaluate the potential role of pyroptosis‐related genes in predicting survival status of HNSCC patients, we conducted Cox regression and LASSO analysis, and identified seven‐gene signature (*CASP1, GSDME, IL6, NLRP1, NLRP2, NLRP6*, and *NOD2*) and built a HNSCC risk model. A significant survival difference was observed between low‐ and high‐risk subgroups of HNSCC patients. Additionally, the risk model was verified in GEO dataset, and it worked well. However, limited patient data were contained in GEO dataset, we need to further verify our model when more relevant data become available. Of note, CASP1 is an essential cysteine protease protein which induces pyroptosis in response to pathological stimuli. After activation, CASP1 cleaves N‐terminal of GSDMD, and allowing its transportation to cell membrane to form pores. *CASP1* gene is identified as a prognostic factor for breast cancer, hepatocellular carcinoma, and pancreatic cancer, and it may influence tumor checkpoint inhibition by assisting T‐cell immunity regulation.^23^ In HNSCC, we found that *CASP1* is upregulated in tumor tissues (Table S2, *p* < .01), and it seemed to be a bad prognostic biomarker (HR > 1), indicating CASP1 might be a tumor‐promoting gene in HNSCC. GSDME is another pore forming molecule which is activated in caspase‐3‐mediated pyroptosis, and its methylation is a potential biomarker in breast cancer. In addition to methylation, the expression of GSDME is positively correlated with a better prognosis in squamous esophageal cancer, while no difference of GSDME expression was observed between tumor and normal tissues in some other cancers.^24^, ^25^ It is difficult to draw a uniform conclusion about the biomarker role of GSDME expression. We found GSDME expression is high‐regulated in HNSCC tumor tissues (Table S2, *p* < .001), and considered it as a poor prognostic biomarker (HR > 1). IL6 is one of the pro‐inflammatory cytokines which is secreted by various types of cells including cancer cells, and it is involved in regulating proliferation and differentiation of cancer cells, and found to be high in serum or tumor tissues of various cancers, such as breast cancer, prostate cancer, pancreatic cancer, etc. Inhibiting IL6 downstream signal pathway may augment therapeutic efficacy in those cancers with elevated level of IL6.^26^, ^27^ Our findings in HNSCC distinguished IL6 as a good prognostic biomarker, but lower expression level of IL6 was detected in tumor tissues compared to normal tissues (Table S2). This is probably due to the secretion of IL6, further investigation of TME is needed. Another bad prognostic biomarker we screened is *NLRP2* gene. NLRP2 belongs to the NACHT leucine‐rich repeat (NLR) family that plays a crucial role in inflammasomes. Common variants in *NLRP2* gene are strongly associated with prognosis after stem cell transplantation.^28^ Additionally, previous study has already identified *NLRP2* as one of the signature genes for predicting of survival in HNSCC patients.^29^ Our findings confirmed this result. Besides, we have discovered three good prognostic biomarkers, *NLRP1, NLRP6,* and *NOD2*. NLRP1 was identified as a first NLR‐family protein and its function related to inflammation has been well studied.^30^ NLRP1 is demonstrated to promote melanoma development via improving inflammasome activation,^31^ and overexpression of NLRP1 in breast cancer cells promotes proliferation, tumorigenesis in nude mice.^32^ Inversely, some other researches pointed that NLRP1 inflammasome decreased colitis and colitis‐associated tumorigenesis.^33^ It is likely that the function of NLRP1 differs in various cancers. In our analysis of HNSCC, *NLRP1* gene tended to be a good prognostic biomarker. NLRP6 inhibited gastric cancer development by promoting the ubiquitination of a heat shock protein GRP78^34^ and another study of gastric cancer cells also demonstrate the tumor suppressing role of NLRP6.^35^ The study of hepatocellular carcinoma indicates that NOD2 is an innate immune sensor initiates the immune response against pathogens, and acted as a tumor suppressor by directly activating AMPK pathway.^36^ These results support the potential good prognostic role of NLRP6 and NOD2 identified in our study of HNSCC.

To further clarify the gene functions and pathways in our established risk model, we unrevealed 161 differential expressed genes between the low‐ and high‐risk subgroups. Functional enrichment analysis disclosed these genes were mainly connected to immune response, suggested the participation of pyroptosis in TME regulation. Our ESTIMATE algorithm results suggested more infiltration of immune cells in TME in high‐risk subgroup compared to low‐risk subgroup. Noteworthy, much higher levels of antitumor immune cells (CD8+ T cells, monocytes, and M1 macrophages) were found in high‐risk subgroup. To our knowledge, CD8+ T cells have long been regarded as an essential antitumor lymphocytes for immune defense to eliminate infections malignant cells.^37^ Monocytes fight against infections, help removing dead cells, boost the immune response.^38^ Uncommitted (M0) macrophage is thought to polarize to M1 and M2 macrophage upon various stimuli, and M1 macrophage executes antagonizing tumor function by producing pro‐inflammatory cytokines.^39^ We speculated this might provide a good opportunity to harness the immune cells to fight the cancer cells by applying immune checkpoint inhibitors.^40^ Another report has manifested that head and neck patients with higher frequencies of PD‐1^high^ CD8+ tumor‐infiltrating lymphocytes correlated with significantly worse disease‐free survival.^41^ It is a big challenge that when and how to choose the proper cancer immunotherapy approaches. Furthermore, we have listed the possible connection between pyroptosis‐related signature genes and drug‐sensitivity. Considering little research explaining proptosis mechanisms in HNSCC,^42^, ^43^ more investigations were required.

Our findings revealed the pyroptosis was closely connected to HNSCC, and 21 pyroptosis‐related genes were differentially expressed between tumor and normal tissues. We also built and verified a risk model for HNSCC prognosis using seven pyroptosis‐related signature genes in TCGA and GEO cohorts. Additionally, we described a different gene expression profile between low‐ and high‐risk subgroups on the basis of our model, and verified those genes were associated with tumor immunity. We further discussed the correlations between signature genes and drug sensitivities. Further in vitro and in vivo experiments are needed to elucidate the gene function pathways.

## AUTHOR CONTRIBUTIONS

ZZL, LFS, and NL conceived and designed the study. LS and YYL collected the data. ZZL perform the statistical analysis and created the Figures. NL wrote the manuscript. All authors have read and approved the final version of the manuscript.

## CONFLICT OF INTERESTS

The authors declare that there are no conflict of interests.

## ETHICAL APPROVAL STATEMENT

The data of this study were obtained from the public database and no ethical approval was required.

## Supporting information


**Appendix S1** Supporting Information

**Appendix S1** Supporting Information

**Table S1** List of 33 pyroptosis‐related genes.

**Table S2** Expression of pyroptosis‐related genes in normal and HNSCC tissues.

## Data Availability

All data generated or analyzed during this study are included in this published article and its supplementary information files.
