# Supplementary figures and images for: Identification of pyroptosis‐related gene prognostic signature in head and neck squamous cell carcinoma
Source: Cancer Med. 2022 May 16;11(24):5129–44. doi: 10.1002/cam4.4825 (PMC9761089; doi:10.1002/cam4.4825)

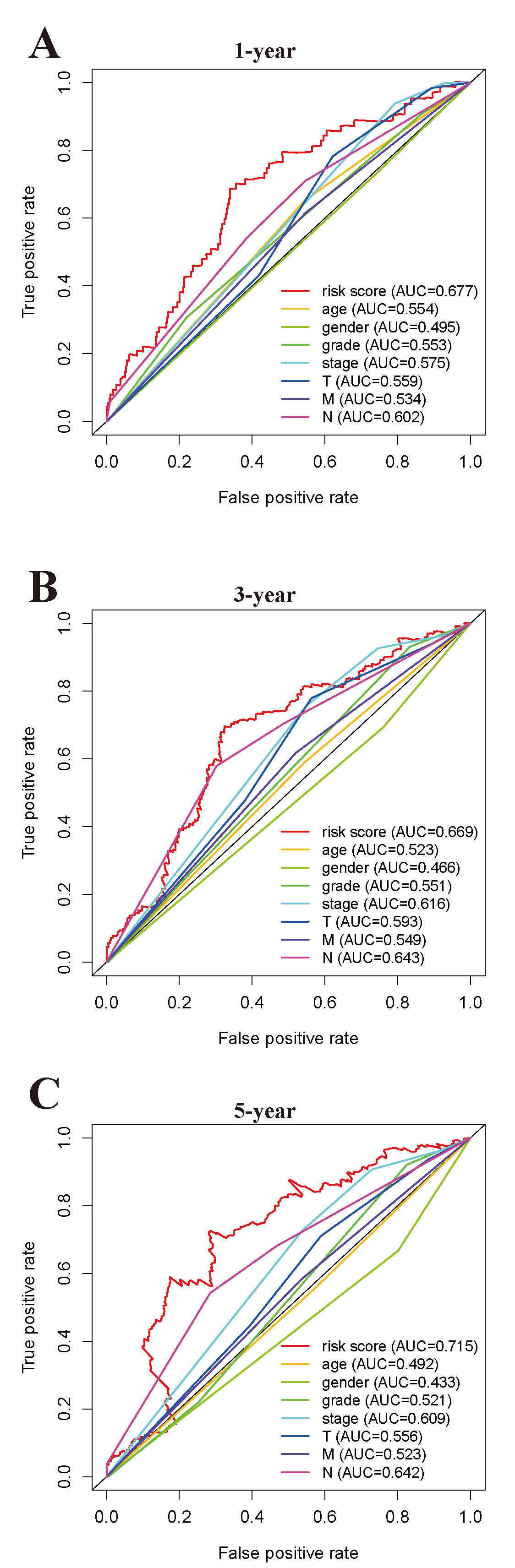

Supplement: Supplementary file 1 — Appendix S1 Supporting Information [file CAM4-11-5129-s002.tif]

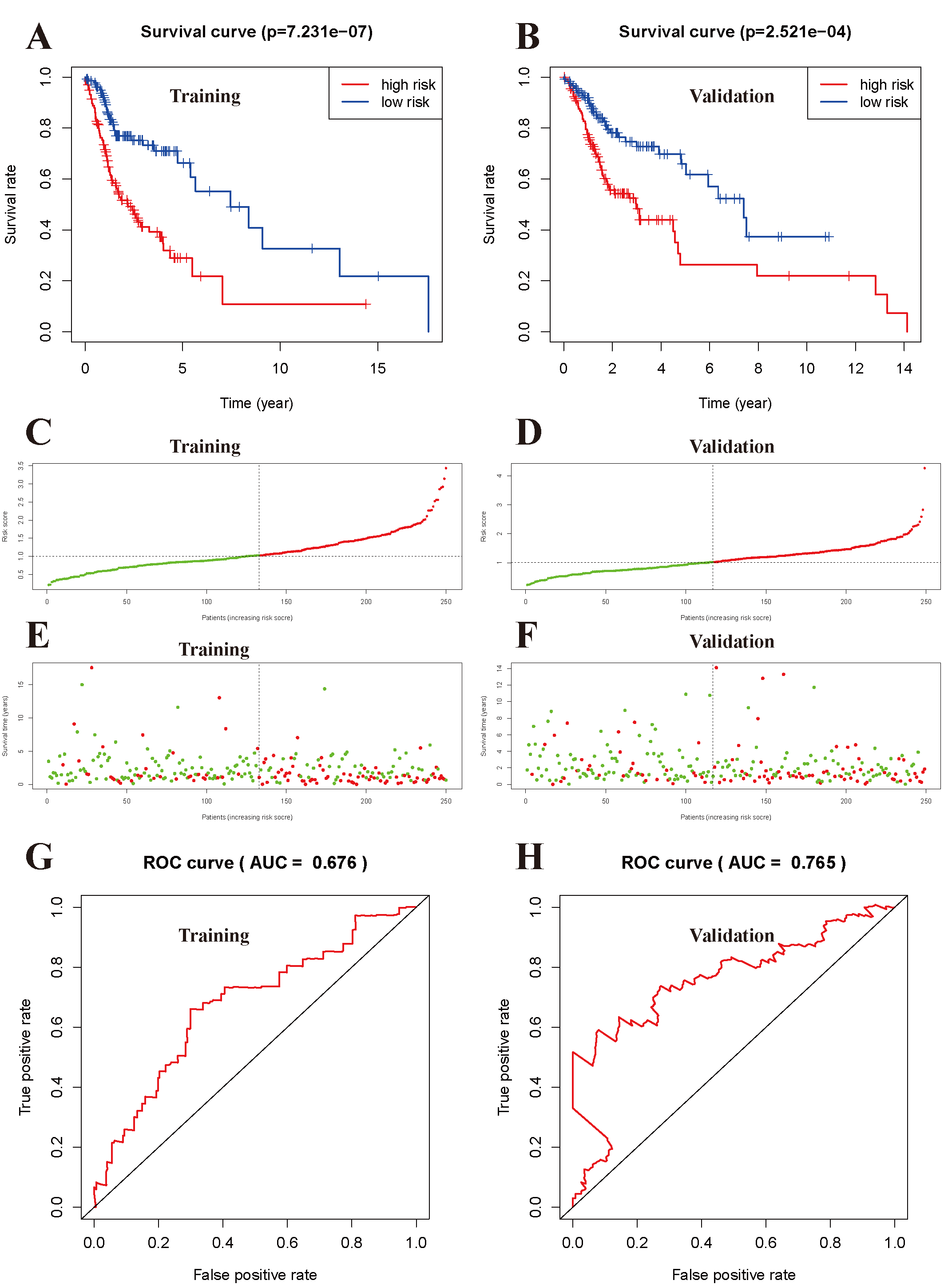

Supplement: Supplementary file 2 — Appendix S1 Supporting Information [file CAM4-11-5129-s003.tif]
